# Supplementary material for: Transcriptional Modulation Reveals Physiological Responses to Temperature Adaptation in Acrossocheilus fasciatus
Source: Int J Mol Sci. 2023 Jul 19;24(14):11622. doi: 10.3390/ijms241411622 (PMC10380296; doi:10.3390/ijms241411622)

Supplementary Material

Supplementary Figure S1. Heat maps of DEGs in different temperature groups and control group , red color in the heat maps’ indicated high expression and blue colors denote indicated low expression. (A) T12 vs T20; (B) T16 vs T20; (C) T24 vs T20; (D) T28 vs T20

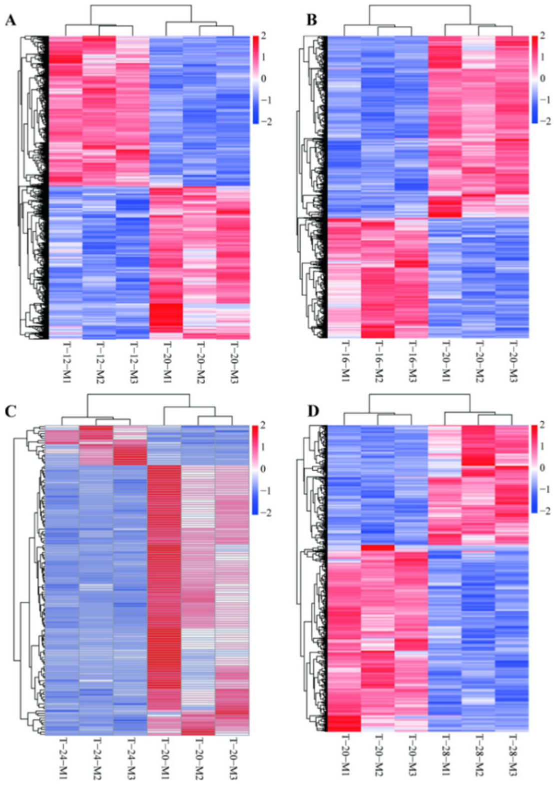

Supplementary Figure S2. Volcano plots of DEGs between different temperature groups and control group, the up-regulated genes by red dots, the down-regulated genes by green dots, and the genes without significant changes in expression by blue dots. (A) T12 vs T20; (B) T16 vs T20; (C) T24 vs T20; (D) T28 vs T20

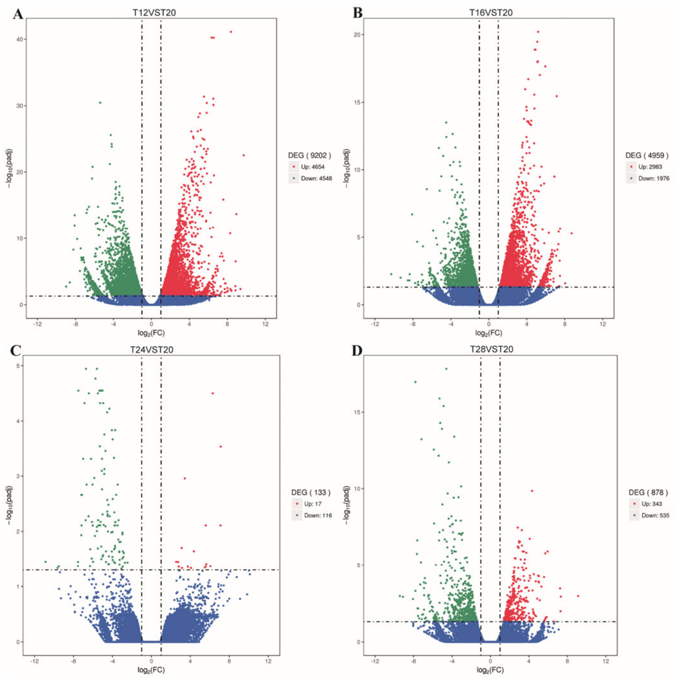

Supplementary Figure S3. The expression of major DEGs of KEGG data in protein digestion and absorption pathways. (*P < 0.05, **P < 0.01, ***P < 0.001)

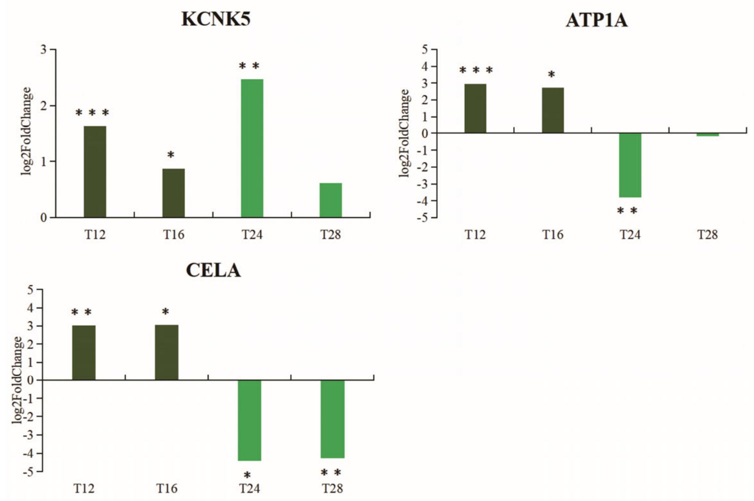

Supplementary Figure S4. The expression of major DEGs of KEGG data in HIF-1 signaling pathway. (*P < 0.05, **P < 0.01, ***P < 0.001)

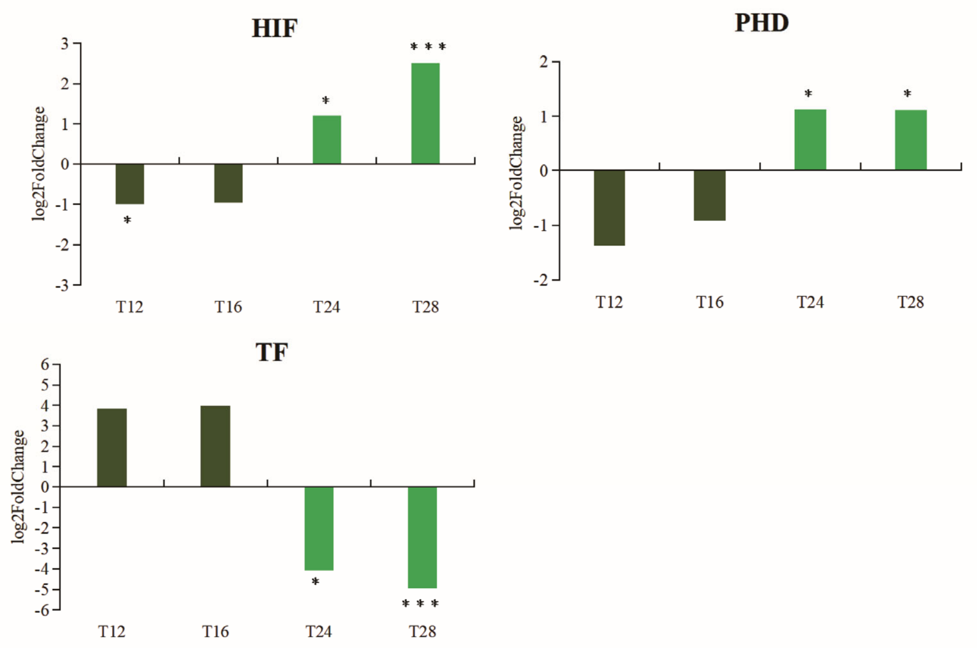

Supplementary Figure S5. The expression of major DEGs of KEGG data in protein PI3K-Akt signaling pathway.(*P < 0.05, **P < 0.01, ***P < 0.001)

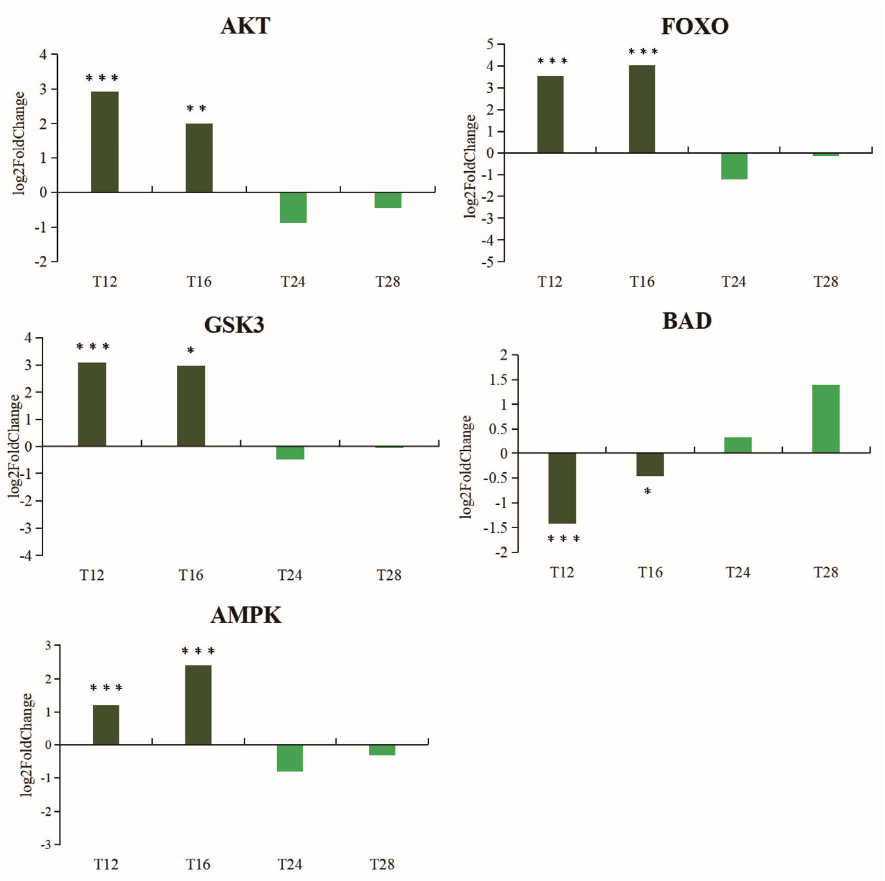

Supplement: Supplementary file 1 [file ijms-24-11622-s001.zip › ijms-2474910-supplementary.pdf]
